# Comparison of renal response to four different induction therapies in Japanese patients with lupus nephritis class III or IV: A single-centre retrospective study

**DOI:** 10.1371/journal.pone.0175152

**Published:** 2017-04-06

**Authors:** Hironari Hanaoka, Tomofumi Kiyokawa, Harunobu Iida, Kana Ishimori, Yukiko Takakuwa, Takahiro Okazaki, Hidehiro Yamada, Daisuke Ichikawa, Sayuri Shirai, Junki Koike, Shoichi Ozaki

**Affiliations:** 1 Division of Rheumatology and Allergology, Department of Internal Medicine, St. Marianna University School of Medicine, Kanagawa, Japan; 2 Division of Nephrology and Hypertension, Department of Internal Medicine, St. Marianna University School of Medicine, Kanagawa, Japan; 3 Department of Pathology, St. Marianna University School of Medicine, Kanagawa, Japan; Keio University, JAPAN

## Abstract

The recent recommendations for the management of lupus nephritis suggest that racial background should be considered while choosing induction therapy. However, the responses to different induction regimens have been poorly studied in Japanese population. Here, we assessed the renal response to different induction therapies in Japanese patients with lupus nephritis class III or IV. The records of 64 patients with biopsy-proven lupus nephritis class III or IV were retrospectively evaluated according to therapy received: monthly intravenous cyclophosphamide (IVCY), the Euro-lupus nephritis trial (ELNT) protocol-IVCY, tacrolimus (TAC), or mycophenolate mofetil (MMF). We investigated cumulative complete renal response (CR) rates and relapse rates for each group for 3 years. Organ damage was assessed with the Systemic Lupus International Collaborating Clinics/American College of Rheumatology Damage Index (SDI). There were 22 patients on monthly IVCY, 18 on ELNT-IVCY, 13 on TAC, and 11 on MMF. Lower systemic lupus erythematosus disease activity index (SLEDAI) and higher CH50 were found in the TAC group at baseline (p<0.01 and p<0.01, respectively). There were no significant differences of cumulative CR rates and relapse free survival for 3 years among the four different therapeutic regimens (p = 0.2 and p = 0.2, respectively). There was a tendency to have early response and early relapse in TAC group and late response in MMF group. The SDI increase over 3 years was found more frequently in the TAC group than in the monthly-IVCY group (p = 0.04). Multivariate analysis indicated that CR at 3 months was independent prognosticator for low damage accrual. Regarding lower damage accrual, early CR achievement might be essential in induction therapy regardless of immunosuppressant choice.

## Introduction

Lupus nephritis (LN) is a common manifestation of systemic lupus erythematosus (SLE) that contributes to significant morbidity and mortality [1]. Although the survival of patients with SLE has improved over the last decades, the 10-year survival rate is still lower than that of age- and sex-matched healthy populations [2]. The most important cause of late mortality is cumulative organ damage [3]. The recent American College of Rheumatology (ACR) guideline for management of LN [4] recommends that patients with biopsy-proven lupus nephritis class III or IV should be treated with either intravenous cyclophosphamide (IVCY) or mycophenolate mofetil (MMF) for induction therapy. The recommendations also state that ethnic and racial background be considered while choosing a therapy. This statement was based on the results of the Aspreva Lupus Management Study (ALMS) [5], which examined the influence of race on renal response to MMF and IVCY. For Black or Hispanic patients, the renal response to MMF was superior to that to IVCY. Asian patients tended to respond better to IVCY than MMF, although a significant difference was not seen. Accordingly, ACR recommended preferential use of MMF protocol for Hispanics and African-Americans [6]. The low dose IVCY regimen was recommended for whites with European background based on the results of Euro Lupus Nephritis Trial (ELNT). In contrast, there have been few investigations of the efficacy and safety of the ELNT protocols focused on other ethnic groups or races, especially Asians [7]. Recently, tacrolimus (TAC) for induction therapy had a good outcome in Chinese patients with lupus nephritis in a short-term study [8]. However, few other reports have evaluated the efficacy of TAC in other Asians, including Japanese [9, 10].

In the present study, we retrospectively analyzed and compared the outcomes of Japanese patients with biopsy proven LN class III or IV to one of the four treatment protocols: monthly IVCY, the ELNT protocol-IVCY, TAC or MMF.

## Materials and methods

### Patients

It was a retrospective study performed on Japanese patients with SLE by ACR classification criteria [11] who had visited St. Marianna University Hospital between 2003 and 2010. Inclusion criteria were class III or IV LN according to the International Society of Nephrology/Renal Pathology Society (ISN/RPS) classification [12]. Patients needed to have received at least 3 years of care in the hospital. We selected patients treated with one of the following immunosuppressive agents as induction therapy: IVCY, low-dose ELNT-IVCY, MMF, or TAC. Of 358 patients, 88 patients had biopsy-proven LN, and 66 had class III or IV and had received one of the four treatment regimens for induction. Two of these were lost to follow-up, leaving 64 patients for final enrollment. Prior approval was obtained from the Ethics Committee of St. Marianna University School of Medicine (approval number 3305). Since this was a retrospective study without any investigation or intervention done besides those for clinical case management by the treating nephrologists, written informed consent was not required in accordance with the guidelines of the Ministry of Health, Labour, and Welfare of Japan.

### Data collection

Information was collected from the hospital records at baseline at the time of renal biopsy before induction therapy and subsequently at 2, 4, 8, 12, 24, 48, 96, and 144 weeks (3 years) after initial induction therapy. Data included demographic features, clinical information, treatment regimens, SLE disease activity index (SLEDAI) [13] and investigation reports. We used the Systemic Lupus International Collaborating Clinics/American College of Rheumatology Damage Index (SDI) to assess systemic damage accrual [14]. We further analyzed corticosteroid-related damage or not corticosteroid-related damage according to the previous report [15].

### Definition of terms

Complete renal response (CR) was defined based on the Joint European League Against Rheumatism and European Renal Association–European Dialysis and Transplant Association (EULAR/ERA-EDTA) recommendations for LN [16], with CR defined as a urine protein: creatinine ratio (UPCR) of 50 mg/mmol and normal or near-normal (within 10% of normal estimated glomerular filtration rate (eGFR) if previously abnormal) renal function, substituting 0.5 g/gCr for UPCR 50 mg/mmol [16]. Relapse included nephritic and proteinuric flare according to EULAR/ERA-EDTA recommendation [16], with decreasing eGFR by ≥10%, active urine sediment, or increasing UPCR more than 1.0 g/gCr after achieving CR.

### Renal pathology

Patients underwent a renal biopsy before induction therapy. All patients were diagnosed according to the ISN/RPS classification [12] by light microscopy and immunofluorescence analysis. Activity index (AI) and the chronicity index (CI) developed by Austin et al. [17] were scored. Morphological features of the standard AI and CI were evaluated separately, namely endocapillary hypercellularity, polymorphonuclear leukocyte infiltration, karyorrhexis/fibrinoid necrosis, cellular crescents, hyaline deposits, interstitial inflammation, glomerular sclerosis, fibrous crescents, tubular atrophy, and interstitial fibrosis. We measured the percentage of these features in individual patients.

### Statistical analysis

Continuous values are shown as mean ± standard deviation (SD). Differences between the groups were analyzed using the Mann-Whitney U-test or Kruskal-Wallis test for nonparametric data and the chi-squared or Fisher’s exact test for categorical data. Cumulative CR rates were calculated using the Kaplan-Meier method, and differences between the two groups were tested with a log-rank test. To identify independent parameters that predict increasing SDI over 3 years defined as more than 1-point increase from baseline, we performed multivariate analysis using initial characteristics previously reported as predictors for good renal outcome [18, 19] and treatment regimens. We selected SLEDAI and CH50 levels as other covariates in multivariate analysis, since they are significantly differed among the treatment groups at their baseline. We performed multiple logistic regression analysis with baseline eGFR level, SLEDAI, CH50 level, immunosuppressant for induction therapy (monthly IVCY use, ELNT-IVCY use, MMF use, and TAC use), immunosuppressant for maintenance therapy (AZA use, MMF use, and TAC use), CR duration and achievement of CR at 3 months as independent variables for increasing SDI.

## Results

### Baseline clinicopathological characteristics and treatment regimens

The 64 patients were divided into four groups according to their induction therapies. Demographic and clinical features at baseline are shown in Table 1. There were 22 patients on monthly IVCY, 18 on the low-dose ELNT-IVCY protocol, 11 on MMF, and 13 on TAC. Among clinical features at baseline, patients undergoing induction with TAC had a significantly lower SLEDAI (p<0.01), a higher serum CH50 (p<0.01) and relatively lower incidence of arthritis (p = 0.82), serositis (p = 0.50), and cytopenia (p = 0.23). All patients received glucocorticoid therapy at an initial dose of 1.0 mg prednisolone equivalent/kg/day for 2–4 weeks. Glucocorticoids were then tapered by 10% of the last dose or 10 mg, as determined by the attending physician. Prednisolone dose did not differ significantly among the groups (p = 0.1). The low-dose ELNT protocol consisted of IVCY 500 mg every 2 weeks for six courses. In the monthly-IVCY protocol, the dose ranged from 350 mg at 1-month interval for three courses to 1000 mg at 1-month interval for six courses. The dose of monthly-IVCY was reduced in some patients because of safety concerns. MMF was started at an initial dose of 0.25–1.0 g/day (412.1 ± 123.8 mg/day) and gradually increased to 2.0 g/day. The starting dose of TAC was reduced in some patients because of safety concerns (range from 0.5 to 3.0 mg/day). The target trough was set as 5.0 to 10.0 mg/dL and was adjusted to a trough value of serum concentrations. After the final course of IVCY or after six months of induction therapy for TAC and MMF users, patients were switched to one of the following immunosuppressive agents for maintenance therapy: azathioprine (AZA) at 100 mg/day, MMF 1.0g/day, TAC 1.5–3.0 mg/day, or mizoribine 150 mg/day. Cumulative cyclophosphamide dose was significantly higher in the patients in the monthly-IVCY group than in the patients in the low-dose ELNT-IVCY group (3415 ± 830 mg vs 3000 ± 0 mg, respectively p = 0.03). Patients in the low-dose ELNT-IVCY and monthly-IVCY groups mostly changed to AZA (83.3% and 45.5%, respectively), while the TAC and MMF groups were mostly continued on the same drugs as maintenance therapy (90.9% and 92.3%, respectively). There were no remarkable differences among the four groups with regard to renal pathological analysis, including ISN/RPS classification, morphological features of LN, or AI and CI.

**Table 1:** Baseline clinical and renal pathological features of lupus nephritis patients with immunosuppressive therapy.

	Monthly-IVCY (n = 22)	ELNT-IVCY (n = 18)	MMF	TAC	*P*
			(n = 11)	(n = 13)	
Gender, female, n (%)	20 (90.1)	13 (72.2)	10 (90.1)	10 (76.9)	0.41
Age, years	39.6 ± 10.5	41.0 ± 14.7	31.6 ± 8.7	42.2 ± 12.9	0.23
BMI, kg/m^2^	21.7 ± 3.1	21.6 ± 2.6	23.0 ± 2.1	23.0 ± 3.6	0.86
Systolic blood pressure, mmHg	126.1 ± 20.8	136.0 ± 15.7	128.3 ± 15.7	127.4 ± 8.3	0.32
Diastolic blood pressure, mmHg	79.2 ± 16.9	83.7 ± 11.7	77.6 ± 9.2	81.4 ± 7.0	0.62
ARB use, n (%)	13 (59.1)	10 (55.5)	5 (45.5)	5 (38.5)	0.64
Dyslipidemia, n (%)	3 (13.6)	4 (22.2)	3 (27.2)	5 (38.5)	0.44
Diabetes mellitus, n (%)	1 (4.5)	2 (11.1)	0 (0)	1 (7.7)	0.19
Disease duration, years	6.3 ± 6.6	9.4 ± 9.3	4.4 ± 6.3	9.7 ± 9.4	0.38
SLEDAI	17.9 ± 3.8	14.9 ± 3.7	16.8 ± 5.0	12.3 ± 5.8	<0.01*
Extra-renal involvement					
CNS, n (%)	1 (4.5)	0 (0)	0 (0)	0 (0)	0.56
Arthritis, n (%)	4 (18.2)	5 (27.8)	2 (18.2)	2 (15.4)	0.82
Skin, n (%)	2 (9.1)	3 (16.7)	0 (0)	3 (23.1)	0.33
Serositis, n (%)	3 (13.6)	2 (11.1)	2 (18.2)	0 (0)	0.50
Cytopenia, n (%)	18 (81.9)	15 (83.3)	10 (90.9)	8 (61.5)	0.23
SDI	0.4 ± 0.7	0.9 ± 0.6	0.2 ± 0.4	0.3 ± 0.4	0.17
Proteinuria, g/gCr	2.6 ± 1.5	3.5 ± 2.1	3.3 ± 3.1	2.4 ± 1.8	0.44
Hematuria, n (%)	19 (86.4)	15 (83.3)	11 (100)	9 (69.2)	0.22
Cellular cast, n (%)	4 (18.2)	3 (16.7)	3 (27.3)	3 (23.1)	0.89
eGFR, ml/min/1.73m^2^	67.1 ± 25.6	75.8 ± 27.3	72.2 ± 37.9	76.3 ±3 2.8	0.86
Anti-dsDNA antibody, IU/mL	221 ± 401	268 ± 388	111 ± 138	138 ± 156	0.21
Anti-cardiolipin antibody, IU/mL	35.3 ± 45.6	19.0 ± 24.6	16.2±18.9	10.3 ± 8.2	0.20
Lupus anticoagulant-positive, n (%)	3 (13.6)	3 (16.7)	3 (27.3)	1 (7.7)	0.63
CH50, U/ml	13.6 ± 7.4	14.5 ± 7.3	19.6 ± 7.8	27.6 ± 14.6	<0.01**
Prednisolone, mg/day	43.5 ± 16.4	49.4 ± 11.6	42.7 ± 17.9	36.9 ± 12.9	0.12
Cumulative cyclophosphamide dose, mg	3415 ± 830	3000 ± 0	-	-	0.03
Maintenance therapy					
Azathioprine, n (%)	10 (45.5)	15 (83.3)	0 (0.0)	1 (7.7)	<0.01^§^
MMF, n (%)	8 (36.3)	1 (5.6)	10 (90.9)	0 (0.0)	<0.01^§§^
TAC, n (%)	2 (9.1)	1 (5.6)	1 (9.1)	12 (92.3)	<0.01^¶^
Mizoribine, n (%)	2 (9.1)	1 (5.6)	0 (0.0)	0 (0.0)	0.51
Renal pathological findings					
ISN/RPS classification					
III (A) or III (A/C), n (%)	7 (31.8)	4 (22.2)	2 (18.2)	6 (54.5)	0.43
III (A) or III (A/C) + V, n (%)	1 (4.5)	0 (0.0)	4 (36.3)	2 (18.2)	0.28
IV (A) or IV (A/C), n (%)	10 (45.5)	9 (50.0)	2 (18.2)	4 (30.8)	0.30
IV (A) or IV (A/C) + V, n (%)	4 (18.2)	5 (27.8)	3 (27.3)	1 (7.7)	0.59
Endocapillary hypercellularity, %	57.2 ± 31.3	36.9 ± 34.7	48.2± 9.8	34.6 ± 26.1	0.42
Leukocyte infiltration, %	2.0 ± 3.5	2.1 ± 5.8	4.1 ± 7.2	1.5 ± 4.1	0.56
Subendothelial hyaline deposits, %	19.1 ± 23.8	34.7 ± 31.4	44.2 ± 38.4	30.7 ± 34.4	0.66
Fibrinoid necrosis/karyorrhexis, %	11.3 ± 15.3	1.3 ± 3.7	8.3 ± 7.6	10.2 ± 16.7	0.14
Cellular crescents, %	10.0 ± 29.3	6.9 ± 10.6	7.2 ± 7.4	2.8 ± 5.5	0.36
Interstitial inflammation, %	1.3 ± 3.5	3.3 ± 8.9	2.2 ± 4.8	1.2 ± 4.8	0.82
Glomerular sclerosis, %	7.8 ± 10.5	7.6 ± 8.5	10.2 ± 7.2	3.1 ± 8.8	0.67
Fibrous crescents, %	1.6 ± 3.0	2.9 ± 5.9	2.0 ± 1.1	1.7 ± 2.9	0.71
Tubular atrophy, %	6.3 ± 6.9	6.6 ± 7.9	5.3 ± 2.8	2.1 ± 4.0	0.65
Interstitial fibrosis, %	6.3 ± 6.9	7.3 ± 7.7	5.3 ± 2.8	2.1 ± 4.0	0.75
Activity index	6.6 ± 4.7	4.8 ± 3.1	5.7 ± 2.0	4.8 ± 2.7	0.65
Chronicity index	1.8 ± 1.7	1.9 ± 1.7	2.2 ± 1.5	1.0 ± 1.4	0.52

Values for treatment groups indicate mean ± standard deviation and remaining values represent n (%). ARB, AngiotensinII Receptor Blocker; SLEDAI, Systemic Lupus Erythematosus Disease Activity Index; CNS, Central Nerve system; SDI, Systemic Lupus International Collaborating Clinics/American College of Rheumatology Damage Index; dsDNA, double stranded DNA; IVCY, intravenous cyclophosphamide; ELNT, Euro-lupus nephritis trial; MMF, mycophenolate mofetil; TAC, tacrolimus

*TAC vs monthly-IVCY, p<0.01; TAC vs MMF, p<0.01.

**TAC vs monthly-IVCY, p<0.01; TAC vs ELNT-IVCY, p = 0.01

§ELNT-IVCY vs TAC, p = 0.01; ELNT-IVCY vs MMF, p<0.01

§§MMF vs TAC, p<0.01; MMF vs ELNT-IVCY, p<0.01

¶TAC vs monthly-IVCY, p<0.01; TAC vs ELNT-IVCY, p<0.01, TAC vs MMF, p<0.01.

### Cumulative CR rates and CR status at year 3

**Fig 1:**
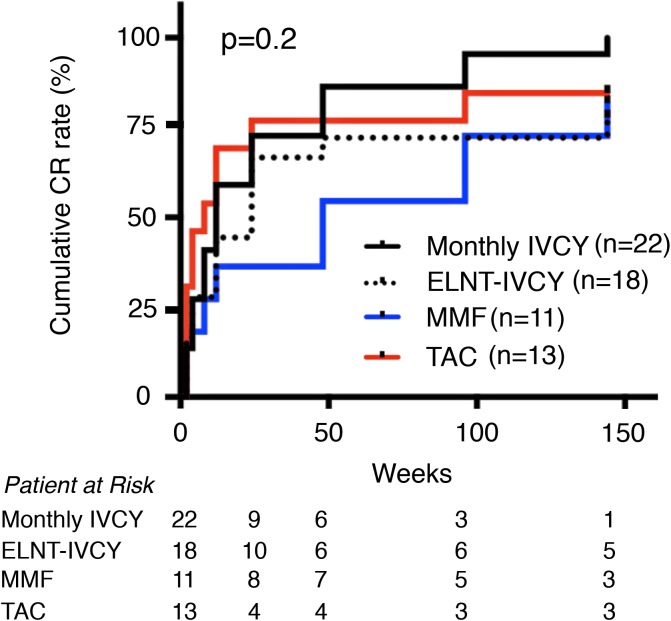
Cumulative complete renal response rate for 3 years after induction therapy. Cumulative complete renal response rate is not significantly different among the four treatment groups (p = 0.2). CR, Complete renal response; IVCY, intravenous cyclophosphamide; ELNT, Euro-Lupus Nephritis Trial; TAC, Tacrolimus; MMF, mycophenolate mofetil.

**Fig 2:**
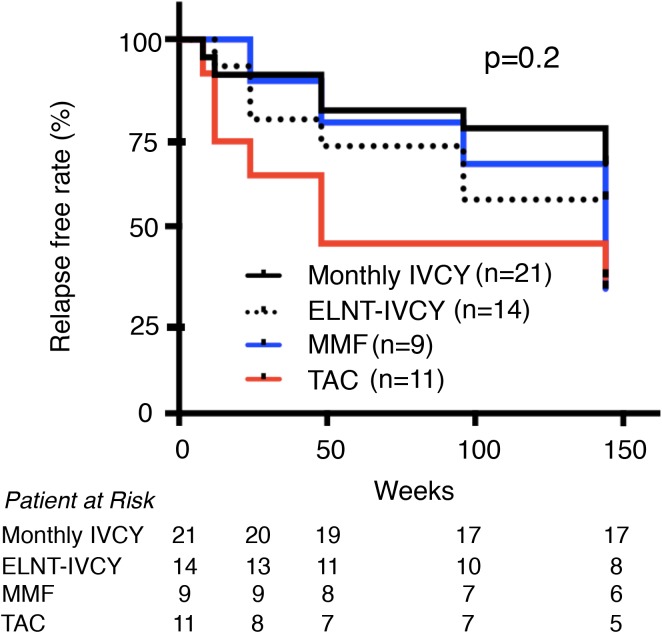
Relapse free rate for 3 years from study enrollment. There was no significant difference among the four treatment groups. CR, Complete renal response; IVCY, intravenous cyclophosphamide; ELNT, Euro-Lupus Nephritis Trial; TAC, Tacrolimus; MMF, mycophenolate mofetil.

**Fig 3:**
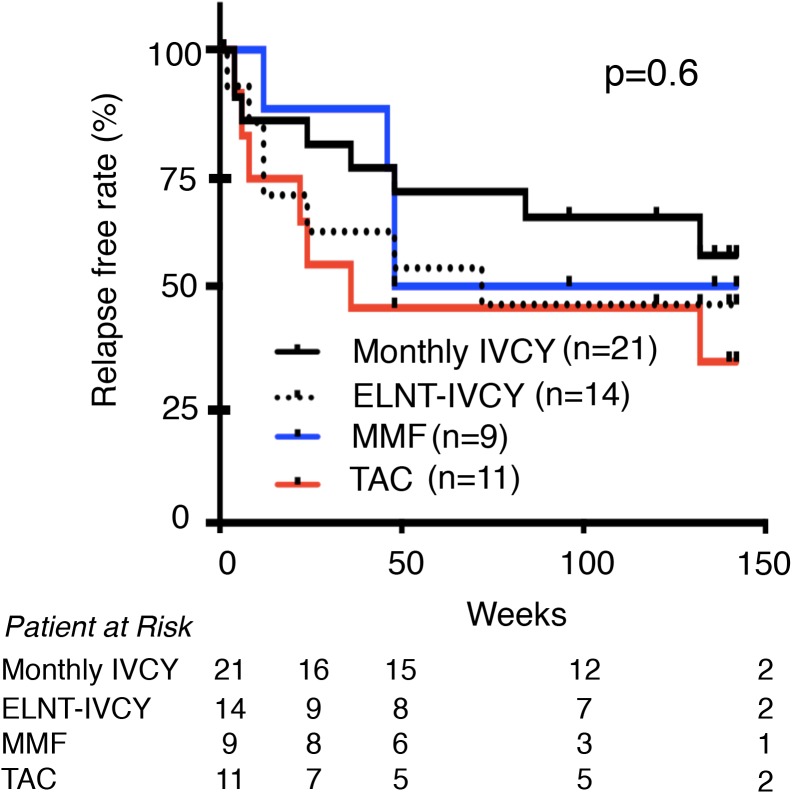
Relapse free rate for 3 years after CR achievement. There was no significant difference among the four treatment groups. CR, Complete renal response; IVCY, intravenous cyclophosphamide; ELNT, Euro-Lupus Nephritis Trial; TAC, Tacrolimus; MMF, mycophenolate mofetil.

We next focused on renal response. Fig 1 shows cumulative CR rates of the four treatment groups. Cumulative CR rates over 3 years were not significantly different among the four groups (monthly-IVCY 95.5%, low-dose ELNT-IVCY 77.8%, MMF 81.8%, and TAC 84.6%, p = 0.2). However, renal response was relatively earlier with TAC and later with MMF compared with the IVCY groups. In IVCY group, there was almost the same renal response by 48 weeks between monthly and ELNT-IVCY but CR rate of monthly-IVCY kept to increase for 3 years compared with ELNT-IVCY. We further investigated the relapse free rate over 3 years from the study enrolment (Fig 2) and the time CR achievement (Fig 3), and found no significant differences among the four groups (p = 0.2 and p = 0.6, respectively). Interestingly, patients with TAC displayed an early renal response for the first year after the induction therapy, but they showed an early relapse.

### Damage accrual and immunosuppressive therapies

**Fig 4:**
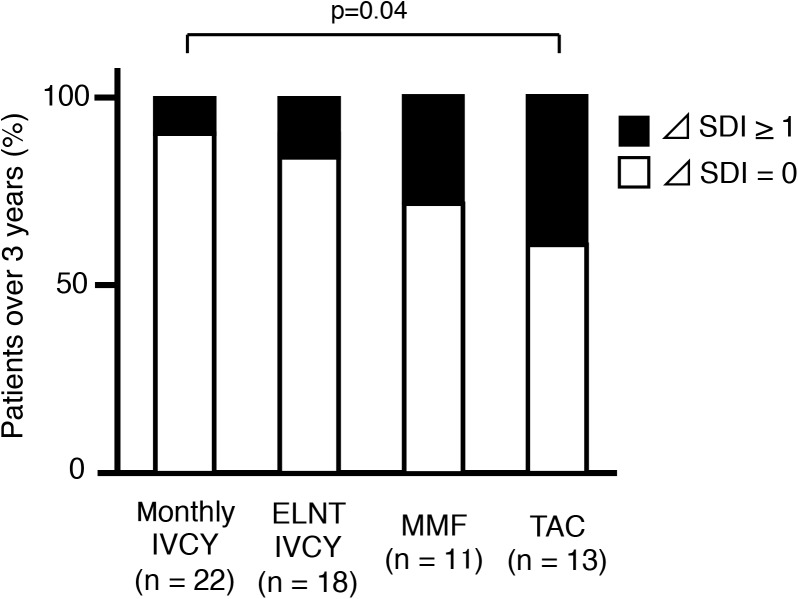
Percentage of patients with increasing damage accrual for 3 years. A higher percentage of patients on TAC had increasing SDI for 3 years compared to those on monthly IVCY (p = 0.04). Montly-IVCY vs ELNT-IVCY, p = 0.55; ELNT-IVCY vs MMF; p = 0.11; MMF vs TAC, p = 0.63; monthly-IVCY vs MMF, p = 0.10, ELNT-IVCY vs TAC, p = 0.32. SDI, Systemic Lupus International Collaborating Clinics/American College of Rheumatology Damage Index; IVCY, intravenous cyclophosphamide; ELNT, Euro-Lupus Nephritis Trial; TAC, Tacrolimus; MMF, mycophenolate mofetil.

**Fig 5:**
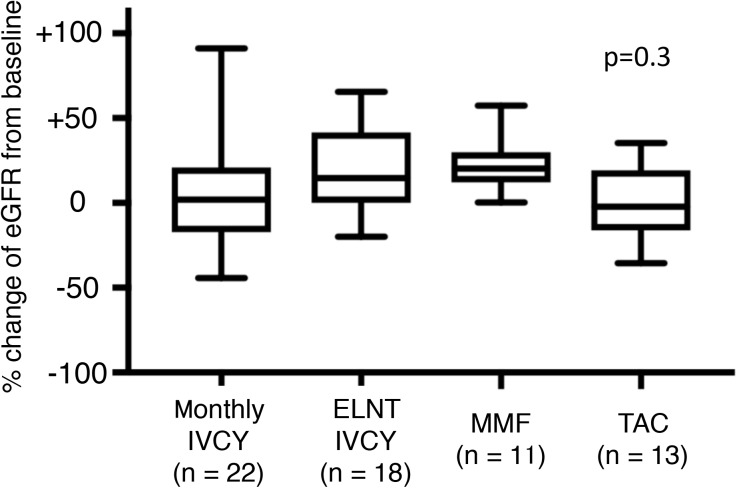
% Change of eGFR from baseline. There was statistically no significant difference among 4 groups (p = 0.3). IVCY, intravenous cyclophosphamide; ELNT, Euro-Lupus Nephritis Trial; TAC, Tacrolimus; MMF, mycophenolate mofetil; eGFR, estimated glomerular filtration rate.

**Fig 6:**
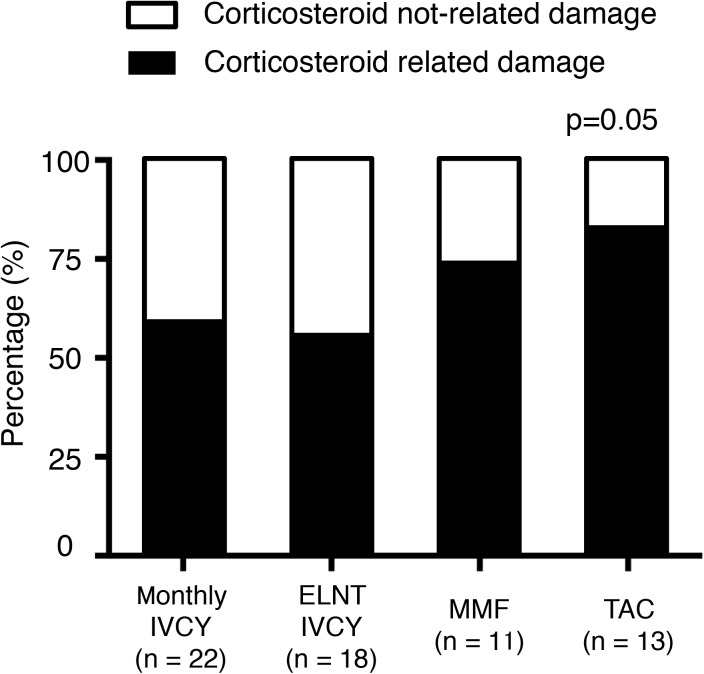
Component of SDI and renal damage at 3 years. Percentage of corticosteroid-related or not corticosteroid-related damage of SDI in 4 groups was shown (p = 0.05). TAC group has higher corticosteroid-related damage than IVCY groups.

**Fig 7:**
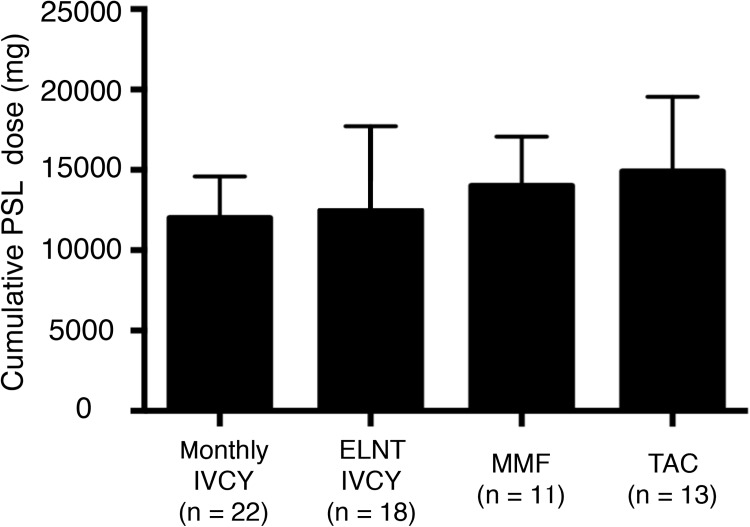
Cumulative PSL dose for 3 years. Cumulative PSL dose was shown. There was no significant difference among 4 groups (p = 0.53) but patients with TAC tended to have a higher cumulative dose of PSL. PSL, prednisolone; IVCY, intravenous cyclophosphamide; ELNT, Euro-Lupus Nephritis Trial; TAC, Tacrolimus; MMF, mycophenolate mofetil.

We analysed SDI over 3 years among the four groups (Fig 4). The lowest damage accrual was found in patients on monthly IVCY. A higher percentage of patients with increasing damage accrual was seen in the TAC group compared with the monthly IVCY group (p = 0.04). We further analyzed the component of the damage. Fig 5 shows % change of eGFR from baseline and Fig 6 shows corticosteroid-related damage or not corticosteroid-related damage. Although there was statistically no significant difference in renal damage, TAC group tended to have higher percentage of corticosteroid-related damage (p = 0.05). Cumulative PSL dose for 3 years were compared among 4 groups (Fig 7). Relatively higher dose of PSL was found in TAC and MMF group without significant difference (monthly-IVCY, 12,002 ± 2,593 mg; ELNT-IVCY, 12,744 ± 3,983 mg; MMF, 14,013 ± 3,069 mg; TAC, 14,898 ± 4,653 mg).

SDI, Systemic Lupus International Collaborating Clinics/American College of Rheumatology Damage Index; IVCY, intravenous cyclophosphamide; ELNT, Euro-Lupus Nephritis Trial; TAC, Tacrolimus; MMF, mycophenolate mofetil.

### Identification of prognostic factors for increasing SDI over 3 years

We performed multivariate analysis with baseline eGFR level, SLEDAI, CH50 level, immunosuppressant for induction therapy (monthly IVCY use, ELNT-IVCY use, MMF use, and TAC use), immunosuppressant for maintenance therapy (AZA use, MMF use, and TAC use), CR duration and achievement of CR at 3 months as independent variables for increasing SDI (Table 2). Univariate analysis revealed CR at 3 months, CR duration and IVCY use for induction therapy were selected negative predictor for increasing SDI (odds ratio 0.10, p<0.01; odds ratio 0.36, p = 0.02; and odds ratio 0.13, p = 0.01, respectively). Among them, the CR at 3 months was identified as independent negative predictor for increasing SDI over 3 years (odds ratio 0.21, p = 0.05) by multivariate analysis.

**Table 2:** Multivariate analysis for predictors of patients with increasing SDI over 3 years.

Parameters	Univariate		Multivariate	
	OR (95% CI)	*P*	OR (95% CI)	*P*
CR at month 3	0.10 (0.02–0.73)	<0.01	0.21 (0.30–0.71)	0.05
CR duration (weeks)	0.36 (0.07–0.89)	0.02	0.61 (0.42–1.01)	0.10
eGFR (ml/min/1.73 m^2^)	1.01 (0.99–1.82)	0.42	-	-
SLEDAI	0.96 (0.94–1.98)	0.56	-	-
CH50 (U/ml)	0.99 (0.95–1.38)	0.40	-	-
Induction therapy				
Monthly IVCY use	0.13 (0.06–0.73)	0.01	0.33 (0.52–0.84)	0.23
ELNT-IVCY use	0.72 (0.15–2.75)	0.65	-	-
TAC use	3.35 (0.84–13.0)	0.08	-	-
MMF use	1.61 (0.31–6.79)	0.54	-	-
Maintenance therapy				
AZA use	0.63 (0.18–2.24)	0.47	-	-
MMF use	0.36 (0.07–1.36)	0.13	-	-
TAC use	1.44 (0.34–5.35)	0.59	-	-

OR, Odds ratio; CI, Confidence interval; SDI, Systemic Lupus International Collaborating Clinics/American College of Rheumatology Damage Index; CR, complete renal response; SLEDAI, Systemic Lupus Erythematosus Disease Activity Index; IVCY, Intravenous cyclophosphamide; ELNT, Euro-lupus nephritis trial; TAC, Tacrolimus; MMF, Mycophenolate mofetil.

### Adverse events

Table 3 summarizes the adverse events experienced by our patients during 3 years after induction therapy. There was no death during the observational period. Major infective episodes developed in 8 (36.3%), 9 (50,0%), 6 (54.5%), and 3 (23.1%) patient treated with monthly-IVCY, ELNT-IVCY, MMF, and TAC, respectively. Respiratory infections and urinary tract infections were frequently reported. There were 2 cases with hospitalization due to bacterial pneumonia and they both were IVCY users. Cytomegalovirus (CMV) reactivation was common in IVCY users but there was no patient with TAC. A significantly higher incidence of leukopenia and anemia was seen in the IVCY group patients than in the others (p<0.01). The comparison of the frequency of these abnormalities between the monthly IVCY and the low-dose ELNT-IVCY group showed no significant difference (p = 0.3).

**Table 3:** Adverse events during 3 years.

	Monthly-IVCY (n = 22)	ELNT-IVCY (n = 18)	MMF	TAC	*P*
			(n = 11)	(n = 13)	
Death	0	0	0	0	-
Serious adverse event	1 (4.5)	1 (5.6)	0 (0)	0 (0)	0.7
Infection (total)					
Respiratory infection	3 (13.6)	1 (5.5)	1 (9.0)	1 (7.7)	0.8
Urinary tract infection	1 (4.5)	3 (16.7)	2 (18.2)	2 (15.4)	0.6
CMV infection	5 (22.7)	5 (27.8)	2 (18.2)	0 (0)	0.2
Gastrointestinal					
Nausea	2 (9.0)	1 (5.6)	2 (18.2)	0 (0)	0.4
Diarrhea	2 (9.0)	0 (0)	0 (0)	0 (0)	0.3
Appetite loss	3 (13.6)	1 (5.6)	2 (18.2)	1 (7.7)	0.7
Laboratory data					
Leukopenia, WBC count < 4,000/μl	11 (50.0)	11 (61.1)	3 (27.2)	1 (7.7)	<0.01*
Anemia, Hb < 11.0g/dl	11 (50.0)	10 (55.6)	2 (18.2)	0 (0)	<0.01**
Thrombocytopenia, Plt count < 10×10^4^/μl	7 (31.9)	4 (22.2)	1 (9.0)	1 (7.7)	0.5
Abnormal liver function test	5 (22.7)	6 (33.3)	2 (18.2)	2 15.4)	0.7

All values for treatment groups indicate n (%). AE, Adverse event; CMV, Cytomegarovirus; WBC, White blood cell; Hb, Hemoglobin; Plt, Platelet; IVCY, Intravenous cyclophosphamide; ELNT, Euro-lupus nephritis trial; MMF, Mycophenolate mofetil; TAC, Tacrolimus.

*TAC vs MMF, p<0.01

**TAC vs monthly-IVCY, p<0.01; TAC vs ELNT-IVCY, p<0.01; MMF vs ELNT-IVCY, p = 0.04.

## Discussion

We found no significant differences of cumulative CR rates and relapse free survival for 3 years among the four different therapeutic regimens as induction therapy for LN. However, the percentage of patients with increasing SDI was lower in the monthly IVCY group than in the TAC groups. Sustained CR status at early phase might have contributed to the low damage accrual in Japanese patients with LN class III or IV.

According to the ACR recommendations for LN management, induction therapy with either IVCY or MMF should be instituted for patients with LN class III or IV [4]. Since treatment response varies depending on location or race/ethnicity, the treatment should be carefully selected [20]. Although many studies have conducted to evaluate the clinical efficacy of immunosuppressant in other populations [5, 6, 8], renal response to immunosuppressant available in clinical practice has been rarely investigated in Japanese. To our best of knowledge, this is the first report comparing renal response to four different immunosuppressants in the population. Among the four therapeutic arms, we found no statistically significant difference in cumulative CR rate and relapse free rate over 3 years. However the renal response varies depending on each immunosuppressant and that might contribute to the difference of damage accumulation.

According to the ALMS trial, which was a multinational study, the renal response to MMF induction therapy was similar to that to IVCY for 6 months [5]. However, as for Asian patients, there was a tendency to have better renal response to IVCY than to MMF while no significant difference was found [5]. Our findings also support these results. Six months after induction therapy, the renal response to MMF tended to be worse than that to IVCY (p = 0.1). However, as the cumulative CR rate on MMF slowly increased with time, it finally reached almost the same rate as that of the IVCY groups over 3 years. Since this study was conducted retrospectively, a degree of selection bias might have been present. Patients with MMF group had higher prevalence of class V and chronicity index and starting dose of MMF was lower than that of internationally accepted dose (412.1 ± 123.8 mg/day). Avoiding ovarian failure, attending physician tended to use MMF for young women. These findings may influence the slow renal response in this study. Furthermore, genetic background for metabolizing MMF may also affect the result. In the pharmacokinetics of MMF, MMF is converted to mycophenolic acid (MPA) presystemically after oral administration, and is then metabolized to mycophenolic acid glucuronide (MPAG). MPAG is then converted to MPA in the enterohepatic circulation; thus, the concentration of MPAG is one of the key factors influencing the concentration and efficacy or safety of MMF [21]. Miura et al. recently analysed the polymorphisms of transporters involved in the excretion of MPAG in Japanese renal transplant recipients. They found that its pharmacokinetics were influenced by organic anion-transporting polypeptide (gene SLCO1B1 and SLCO1B3) polymorphisms [21]. These genetic backgrounds might influence the plasma concentration of MPAG in Japanese patients, resulting in the slow renal response.

TAC is an important alternative agent for LN and has been successfully used in membranous subtype or refractory disease [22–24]. In clinical practice in Japan, as in this study, patients with mild disease course including low disease activity and low incidence of extra-renal involvement were selected to use this agents. Recently, Mok et al. conducted an open randomized controlled study to compare the efficacy of TAC and MMF in Chinese LN patients [8] At 6 months, the CR rate was similar between the two groups, although renal flare was relatively more common in the TAC group. Since nearly 20% of the enrolled patients had class V and the dose of TAC was higher (0.06–0.1mg/kg/day) than in our study, it may be difficult to fully compare with our results. Patients receiving TAC had an early renal response and early relapse in the first year after induction therapy compared with the other groups. Our results not only support those of Mok’s study but also suggest that TAC is suitable for induction treatment within 1 year, but is less effective as a maintenance therapy. As shown above, since enrolled patients in this study were relatively mild disease course, this clinical difference may influence the early renal response. Therapeutic potential of TAC needs to be further investigated.

This is also the first report comparing the clinical efficacy between monthly IVCY and low-dose ELNT-IVCY in Japanese patients with LN class III or IV. While a favourable clinical course was obtained in patients treated with either regimen, a better clinical response was observed in monthly IVCY group. Baseline clinical characteristics were not significantly different between the two groups. However, a higher cumulative dose of cyclophosphamide was received by the monthly IVCY group. Since low-dose ELNT-IVCY has been mainly evaluated in European patients [6], a higher dose of cyclophosphamide might be needed for Asians. Rathi M et al. recently reported, in a head-to-head trial comparing ELNT-IVCY and MMF in an Indian population, that the CR rate at 6 months was not significantly different between the two groups [7]. Further investigation is needed regarding appropriate dosage of IVCY in Asians, especially the Japanese population. Whole incidence of adverse events was not statistically different between monthly IVCY and ELNT-IVCY groups. But we found the incidence of respiratory infection and gastrointestinal adverse events were slightly increased in patients with monthly IVCY. Lower level of eGFR at baseline might influence the higher incidence rate of adverse event, although not significant. A longer-term observation is needed to confirm the precise incidence of other adverse events including ovarian failure and malignancy between these groups.

Our results suggest CR status at early phase may predict low damage accrual. Our findings are supported by a previous study by Rahman et al., who noted that early renal damage correlated with future damage accrual [25]. Several previous reports investigated renal outcomes in patients with lupus nephritis class III or IV by focusing on renal responses [19, 26–29]. These indicate that a sustained complete response and early renal response are associated with reduced long-term mortality. Recently, Joo et al. demonstrated LN is associated with increased corticosteroid-associated damage compared with non-LN [30]. It is also very important to aim for the lowest corticosteroid dose needed to control disease activity, especially for patients with LN.

In adverse event, IVCY users had a significantly higher incidence of cyopenia and slightly common bacterial infection and CMV reactivation. Since it has been retrospectively analyzed, information of previous vaccinations was lacking among the groups. Overall, there was no deceased patient but 2 patients were hospitalized due to bacterial pneumonia.

We could find some strengths of this study. Early CR achievement might be essential for reducing systemic damage regardless of immunosuppressant choice in Japanese patients with lupus nephritis. For achieving early CR, starting dose should be reconsidered and therapeutic drug monitoring for mycophenolic acid might be needed [31] in MMF users and quick change of immunosuppressant from TAC to another by 3 to 6 months after induction therapy may be needed in TAC users. Furthermore, IVCY might be good choice for early achievement of CR and better to choose monthly than ELNT-protocol for efficacy but safety concern should be taken in consideration.

The present study is limited by its single-centre, retrospective design, relatively short observational period and small sample size, which limited the significance of some findings. The differences in disease findings and intensity of therapy among the groups may have failed to show statistical significance, owing to the small sample size. The baseline clinical characteristics in the sample were quite variable. Because this study was retrospectively conducted, patients with lower disease activity were selected in TAC's therapy. These limitations may make our findings less convincing. Furthermore, since the patients were all Japanese, our data may not be comparable to other ethnic groups. Renal response might differ depending on the ethnic and racial background. Therefore, a multi-center, prospective study is definitely required to confirm our findings.

In conclusion, this retrospective study found that the cumulative CR rate for 3 years was not significantly different among the four different therapeutic-regimen groups while the SDI was significantly increased in patients treated with TAC. Sustained CR status for early phase might contribute to low damage accrual at least in the Japanese patients with lupus nephritis.
